# Malaria Exposure in Ann Township, Myanmar, as a Function of Land Cover and Land Use: Combining Satellite Earth Observations and Field Surveys

**DOI:** 10.1029/2020GH000299

**Published:** 2020-12-01

**Authors:** Amanda Hoffman‐Hall, Robin Puett, Julie A. Silva, Dong Chen, Allison Baer, Kay Thwe Han, Zay Yar Han, Aung Thi, Thura Htay, Zaw Win Thein, Poe Poe Aung, Christopher V. Plowe, Myaing Myaing Nyunt, Tatiana V. Loboda

**Affiliations:** ^1^ Department of Geographical Sciences University of Maryland College Park MD USA; ^2^ School of Public Health, Maryland Institute for Applied Environmental Health University of Maryland College Park MD USA; ^3^ Department of Medical Research Myanmar Ministry of Health and Sports Yangon Myanmar; ^4^ National Malaria Control Programme Myanmar Ministry of Health and Sports Naypyitaw Myanmar; ^5^ Duke Global Health Institute Myanmar Program Yangon Myanmar; ^6^ Duke Global Health Institute Duke University Durham NC USA

**Keywords:** malaria, remote sensing, land cover, land use, Myanmar

## Abstract

Despite progress toward malaria elimination in the Greater Mekong Subregion, challenges remain owing to the emergence of drug resistance and the persistence of focal transmission reservoirs. Malaria transmission foci in Myanmar are heterogeneous and complex, and many remaining infections are clinically silent, rendering them invisible to routine monitoring. The goal of this research is to define criteria for easy‐to‐implement methodologies, not reliant on routine monitoring, that can increase the efficiency of targeted malaria elimination strategies. Studies have shown relationships between malaria risk and land cover and land use (LCLU), which can be mapped using remote sensing methodologies. Here we aim to explain malaria risk as a function of LCLU for five rural villages in Myanmar's Rakhine State. Malaria prevalence and incidence data were analyzed through logistic regression with a land use survey of ~1,000 participants and a 30‐m land cover map. Malaria prevalence per village ranged from 5% to 20% with the overwhelming majority of cases being subclinical. Villages with high forest cover were associated with increased risk of malaria, even for villagers who did not report visits to forests. Villagers living near croplands experienced decreased malaria risk unless they were directly engaged in farm work. Finally, land cover change (specifically, natural forest loss) appeared to be a substantial contributor to malaria risk in the region, although this was not confirmed through sensitivity analyses. Overall, this study demonstrates that remotely sensed data contextualized with field survey data can be used to inform critical targeting strategies in support of malaria elimination.

## Key Points

- Villages in Ann Township, Myanmar, exhibiting high forest cover are strongly associated with increased risk of malaria
- Ann Township, Myanmar, villagers living in villages where croplands are the dominant land cover type experience decreased malaria risk
- Remote sensing offers a means to locate LCLU areas associated with high malaria risk to allow for more efficient targeted interventions

## 1 Introduction

Despite considerable progress toward elimination in the past decades, malaria remains a significant global public health burden and priority. In 2013 the United Nations released its ambitious Sustainable Development Goals for the year 2030. Goal 3, Target 3.3 directly relates to malaria by setting a goal to “end the epidemics of AIDS, tuberculosis, malaria, and neglected tropical diseases” (Griggs et al., 2013). Similarly, the World Health Organization (WHO) released its own ambitious goal for malaria in 2016, namely, at least a 40% reduction in malaria cases by 2020, at least 75% by 2025, and at least 90% by 2030 (WHO, 2015a). As we approach the first milestone year for WHO's ambitious plan, progress has unfortunately stalled (WHO, 2019). In response, the WHO shifted its priorities to a new aggressive plan titled “High burden to high impact: a targeted malaria response” (WHO, 2019). Four key elements define this new plan, the second of which includes moving away from a “one‐size‐fits‐all” approach and instead using data‐driven methodologies to pinpoint where to deploy the most effective malaria control tools for maximum impact.

Although the number of malaria‐driven deaths is highest in Africa, the urgency of malaria elimination is equally high in Southeast Asia, where there has been a documented emergence of Artemisinin‐resistant *Plasmodium falciparum* parasites (WHO, 2015a). For this reason, the WHO also released the “Strategy for malaria elimination in the Greater Mekong Subregion (GMS)” (WHO, 2015b). Since the implementation of this strategy, malaria cases have fallen dramatically across the GMS. However, forward momentum must continue to reach full eradication. The country of Myanmar has been a success story, with a decrease of 82% of malaria cases in the country from 2012–2017 (WHO, 2018). However, the country is facing numerous challenges which could inhibit its forward progress, including artemisinin‐resistant parasites, pyrethroid‐resistant malaria vectors (WHO, 2018), lengthy borders with other malarious countries (Bhumiratana et al., 2013; Kounnavong et al., 2017; Parker et al., 2015), and health care access issues for mobile/migrant populations (NMCP, 2016, 2017).

Following the call from the WHO to pinpoint malaria control for the highest impact and the urgency of keeping momentum in Myanmar, now more than ever, it is critical to find feasible ways to implement targeted intervention strategies. Similar to other low‐transmission areas, malaria prevalence across Myanmar is heterogeneous, patchy, and complex. Further complicating matters is the high prevalence of asymptomatic, low‐density malaria infections (Adams et al., 2015; Imwong et al., 2014, 2015). Using an ultrasensitive reverse transcription PCR (usPCR) assay (Adams et al., 2015), we are finding that the prevalence of malaria at sites within and bordering Myanmar is highly heterogeneous. Villages with high prevalence are often close to villages with little or no malaria. While acute cases are more likely to seek treatment, allowing for easier monitoring and disruption of transmission, asymptomatic carriers are unaware of the need to seek treatment and therefore represent a silent and long‐lasting reservoir that can significantly hinder elimination efforts (Lindblade et al., 2013). Strategies that rely on self‐reporting of infection to track malaria hot spots will be insufficient when seeking to eliminate the last few pools of malaria remaining in the country. While a census level collection of blood samples would be the ideal way to identify these remaining parasite pools, such an undertaking would be extremely costly and challenging to implement. An intermediary that can target likely hot spots of infection is needed to inform the sampling scheme necessary to capture the few remaining malaria reservoirs.

Spatial statistical modeling has been deployed in multiple countries for malaria forecasting (Rogers et al., 2002; Thomson et al., 2006). Such models typically rely on environmental variables that are associated with the habitat suitability and population dynamics of the malaria mosquito vector. When these environmental variables are forecast over space and time, predictive maps of vector densities can be created. However, previous studies have shown that models that rely solely on vector densities are only loosely associated with actual malaria prevalence. Models that incorporate factors relating to human behavior and human population, in conjunction with vector densities, align better with observations of malaria distribution (Mwakalinga et al., 2016; Ngom & Siegmund, 2010).

An example of human behavior associated with malaria is occupation or livelihood. How people live, work, and move through their landscape can increase or decrease their risk of contracting malaria. For example, Zaw et al. (2017) found a higher prevalence of asymptomatic malaria among Myanmar workers with forest‐related occupations, with an odds ratio approximately 10 times greater than study participants whose occupation was not forest related. Soe et al. (2017) found high associations between malaria morbidity and occupation, with fire woodcutters at the highest risk and nighttime rubber tree tappers at the lowest risk. While no country‐wide data sets describing occupational exposure exist and obtaining those data via surveys is prohibitively expensive and frequently not feasible in remote hard‐to‐reach areas, many of the parameters describing potential occupation‐ or livelihood‐related malaria exposure can be captured through satellite‐based land cover and land use (LCLU) mapping. Land cover (LC) describes the physical properties of the landscape (tree, shrub or grass cover, open water, impervious surface, etc.), while land use (LU) describes how humans are using the land in question (plantation, natural forest, built structures, and cropped areas). In combination, LCLU maps can be used as a proxy for human activity on the landscape, allowing for incorporating livelihood exposure metrics into malaria models.

Within Myanmar, the patterns of LCLU are incredibly variable in space and time. Myanmar is a rapidly developing economy that rejoined the global markets relatively recently. Rapid LC change is occurring across the country, with estimates of nearly 20,000 km^2^ of intact forest lost occurring annually (Bhagwat et al., 2017). With such rapid changes occurring, satellite remote sensing offers a methodology for capturing the composition of LCLU while also monitoring the change in the environmental conditions both related to vector dynamics and population exposure.

Moderate‐resolution remote sensing instruments, such as Landsat, are particularly well suited to collect data at scales relevant to human activity, specifically the smaller‐scale patterns of LCLU within remote regions of Myanmar where the majority of malaria pools persist. However, satellites are unable to capture the full scope of how people engage in various LUs. This research seeks to contextualize moderate‐resolution remotely sensed LC data using survey data that questions how people use the land for a remote township within Myanmar. The goal of this research is to define criteria for easy‐to‐implement remote sensing methodologies that can increase the efficiency of targeted malaria elimination strategies.

## 2 Materials and Methods

### 2.1 Study Area

**Figure 1:**
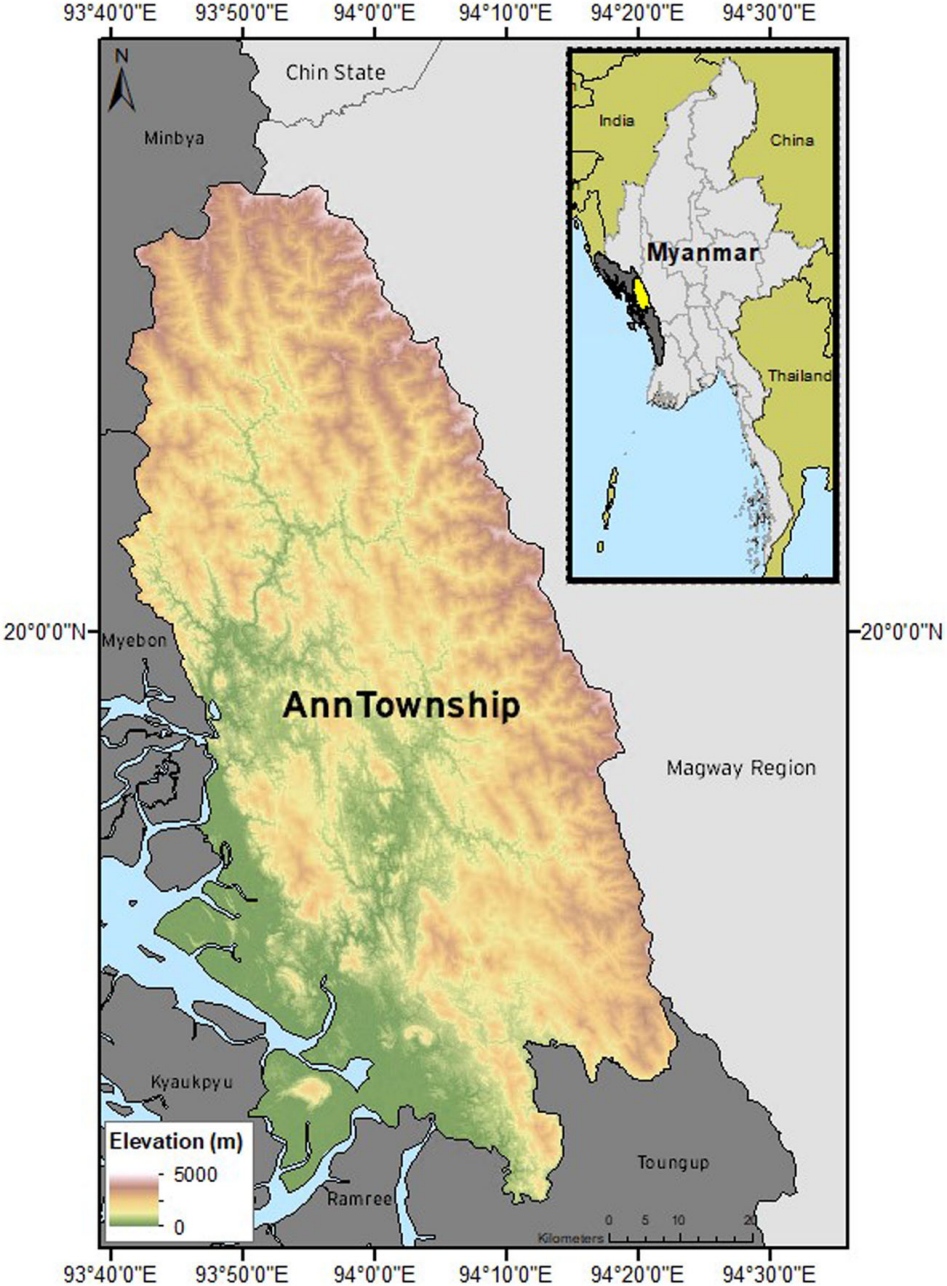
Map detailing the location of the study area: Ann Township, Rakhine State, Myanmar.

Five remote villages dispersed across subtropical Ann Township, an administrative region (similar to a county) within Rakhine State, Myanmar (Figure 1), were surveyed. Ann Township is within the Rakhine Coastal climatic region, which is extremely conducive to mosquito breeding. The region has a warm annual average temperature of 25.2°C and the highest annual precipitation across all of Myanmar, averaging 3,200 mm/year (Horton et al., 2017). Consequently, Rakhine State carries a high malaria burden for the region, with an estimated Annual Parasite Incidence (API) of 9.54 in 1,000 population for any malaria according to the Vector Borne Disease Control Annual Report 2016 compiled by the National Malaria Control Programme (NMCP), Ministry of Health and Sports, Myanmar (NMCP, 2017). This incidence rate is much higher than the estimated API of 0.14 and 0.29 for neighboring regions Magway and Bago, respectively.

Ann Township stretches from the west coast eastward to mountainous terrain. Ann specifically carries a high malaria caseload, in comparison with other GMS locations, and the largest asymptomatic reservoir, detected by sensitive molecular techniques (unpublished data, Nyunt), despite an overall low‐transmission rate. The primary malaria vectors are forest‐dwelling *Anopheles dirus* and foothill and valley‐dwelling *Anopheles minimus* (Oo et al., 2004). Malaria parasites *Plasmodium vivax* and *P*. *falciparum* are most commonly identified in the region; however, *P*. *knowlesi* has been recently identified elsewhere in Myanmar (Ghinai et al., 2017; Jiang et al., 2010).

The population of Ann is dynamic and distributed across the landscape in a highly uneven pattern, with human settlements covering less than 0.1% of the total land area (Hoffman‐Hall et al., 2019). This uneven distribution results in isolated groups of people that can serve as primary drivers of infectious diseases (in this case, malaria) into previously disease‐free regions upon migration or travel (Martens & Hall, 2000). Eliminating malaria from these isolated populations (transmission “sources”) is difficult as NMCP is often unable to reach and engage with them; however, left untreated, these groups could hinder overall elimination progress. The next step, therefore, must be to discover which characteristics, identifiable via remote sensing, influence malaria transmission in these populations to facilitate the quick and efficient identification of areas in need of targeted elimination interventions.

### 2.2 Data

#### 2.2.1 Malaria Prevalence

A prospective cross‐sectional study was conducted in March–June 2016 to estimate the prevalence of malaria in five remote villages in Ann township in Rakhine State (referred to in this study as Villages A, B, C, D, and E). The study was independently reviewed and approved by the Institutional Review Boards of the Myanmar Department of Medical Research, Duke University, and the University of Maryland College Park. The village selection was based on known or suspected malaria burden and research team capability to ensure the integrity of the study data and samples. Inclusion criteria included age at least 6 months old, compliance with study procedure, and written informed consent. Community outreach was conducted twice before the study to ensure community buy‐in and adequate dissemination of knowledge about the upcoming study. The primary means of study recruitment was word of mouth, recognized as effective in previous studies (Huang et al., 2017). A two‐stage household‐ and individual‐based randomization scheme was used to approximate the local population as closely as possible. Households were selected using a random number randomization method, until a target sample size was reached, assuming that each household was composed of 4.5 (ranging between 2 and 12) members on average, based on the census data. From each household, if the household contained four or fewer, everyone was sampled, or four individual members were randomly selected from larger households using a random number method. Written consent was obtained from a designated head of the household and each participant. If a potential participant refused or was unavailable, the next nearest household was selected. Data were collected using a standardized and validated questionnaire (see below) by trained study personnel. Finger prick blood was collected for malaria testing using a rapid diagnostic test (RDT) and transferred on to a filter paper for laboratory molecular analysis using an ultrasensitive polymerase chain reaction (usPCR) method. The result of the RDT was documented in real time. Filter paper blood samples were labeled and air dried. These dried blood spot (DBS) samples were placed in an individual ziplock bag containing desiccant and stored in a refrigerator until transport to a central lab. *P*. *falciparum*, *P*. *vivax*, and mixed infections were all identified by usPCR (Zainabadi et al., 2017).

A total of 990 participants was enrolled and completed the study successfully. No unexpected or severe adverse events were reported. Only one case of *P*. *falciparum* was identified by RDT and was treated by a referred care team, following the national treatment guidelines. The characteristics of the study population, study villages, and village‐based malaria prevalence for *P*. *falciparum* monoinfection, *P*. *vivax* monoinfection, and mixed infection are summarized in Table 1. Those with usPCR‐positive malaria were not treated since treatment is not recommended by the Myanmar national program or the WHO nor is there a clear understanding of a risk‐benefit ratio for treating them.

**Table 1:** Descriptive Statistics of the Sample Population

Village	A	B	C	D	E	Total
Population sampled, *n*	193	198	192	199	197	979
Sex: *n* (% of sample village population)						
Female	96 (49.7%)	108 (54.5%)	93 (48.4%)	0 (0%)	91 (46.2%)	388 (39.6%)
Male	97 (50.3%)	90 (45.5%)	99 (51.6%)	199 (100%)	106 (53.8%)	591 (60.4%)
Age: *n* (% of sample village population)						
0–14	60 (31.1%)	60 (30.3%)	80 (41.7%)	0 (0%)	33 (16.8%)	233 (23.8%)
15–24	33 (17.1%)	27 (13.6%)	34 (17.7%)	24 (12.1%)	34 (17.3%)	152 (15.5%)
25–54	71 (36.8%)	80 (40.4%)	65 (33.9%)	170 (85.4%)	125 (63.5%)	511 (52.2%)
55–64	17 (8.8%)	17 (8.6%)	6 (3.1%)	5 (2.5%)	4 (2.0%)	49 (5.0%)
65+	12 (6.2%)	14 (7.1%)	7 (3.6%)	0 (0%)	1 (0.5%)	34 (3.5%)
Pregnancy status: *n* (% of sample village female population)						
Pregnant	1 (1.1%)	1 (0.9%)	0 (0%)	0 (0%)	4 (4.4%)	6 (1.5%)
Not pregnant (excludes males)	95 (98.9%)	107 (99.1%)	93 (100%)	0 (0%)	87 (95.6%)	382 (98.5)
Travel: *n* (% of sample village population)						
Participant traveled outside village in past 6 months	86 (44.6%)	75 (37.9%)	52 (27.1%)	198 (99.5%)	52 (26.4%)	463 (47.3%)
Family member(s) of participant traveled outside village in past 6 months	112 (58.0%)	115 (58.1%)	83 (43.2%)	39 (19.6%)	41 (20.8%)	390 (39.8%)
Self‐reported exposure: *n* (% of sample village population)						
Frequently visits forest areas	44 (22.8%)	54 (27.3%)	24 (12.5%)	75 (37.7%)	52 (26.4%)	249 (25.4%)
Frequently visits plantations	0 (0%)	3 (1.5%)	24 (12.5%)	109 (54.8%)	67 (34.0%)	203 (20.7%)
Frequent visits farms	79 (40.9%)	91 (46.0%)	91 (47.4%)	14 (7.0%)	8 (4.1%)	283 (28.9%)
Did not select any land use option	107 (55.4%)	100 (50.5%)	99 (51.6%)	74 (37.2%)	112 (56.9%)	492 (50.3%)
Malaria prevalence: *n* (% of sample village population)						
*P*. *falciparum* mono	9 (4.7%)	3 (1.5%)	13 (6.8%)	1 (0.5%)	2 (1.0%)	28 (2.7%)
*P*. *vivax* mono	9 (4.7%)	6 (3.0%)	22 (11.5%)	12 (6.0%)	9 (4.6%)	58 (5.9%)
Mixed *P*. *falciparum* and *P*. *vivax*	1 (0.5%)	1 (0.5%)	4 (2.1%)	0 (0%)	0 (0%)	6 (0.6%)
Any malaria	19 (9.8%)	10 (5.1%)	39 (20.3%)	13 (6.5%)	11 (5.6%)	92 (9.4%)

#### 2.2.2 Data on Malaria Risk

A questionnaire was developed to collect demographic information for each participant, based on malaria literature which commonly identifies the following variables as potential confounders: age (O'Meara et al., 2008), pregnancy status (Desai et al., 2007), travel status (“have you traveled outside of the village in the past six months?”), and family travel status (“has anyone in your family traveled outside the village in the past six months?”) (Wesolowski et al., 2012). Sex was also assessed as a possible confounder and effect modifier (Ayele et al., 2012). Each participant was also surveyed about their prior symptoms to assess the subclinical nature of malaria infection. Prior symptoms included fever, headache, body ache, nausea, vomiting, abdominal discomfort, decreased appetite, or fatigue, all within the previous 2 months, as well as fever within the previous 24 hr. Axillary body temperature of each participant was recorded at the time of data collection.

#### 2.2.3 Exposure: LU, LC, and Forest Cover Change

As part of the questionnaire, participants were asked about their LU in areas relevant to malaria exposure. Specifically, they were asked if they visited any of the following locations frequently (defined as at least twice a week or continuous 2 weeks): farm, forested area, plantation, mine, and refugee camp.

Satellite‐based LCLU data were derived from a 30‐m LCLU map of Ann in 2016 created for this research (Chen et al., 2020). Eight classes were identified in the following order: (1) water, (2) human infrastructure, (3) croplands, (4) managed forest (i.e., plantations), (5) natural forest, (6) topographic depressions, (7) shrub and grass, and (8) bare ground. The water class was mapped using the Landsat Surface Water Fraction algorithm (DeVries et al., 2017). Human infrastructure was a combination of impervious surface mapped by the Global Man‐made Impervious Surface (GMIS) data product (Brown de Colstoun et al., 2017) and Ann Township villages mapped at 30‐m resolution by Hoffman‐Hall et al. (2019). The croplands class was mapped using the Global Food Security‐support Analysis Data (GFSAD) Cropland Extent 30‐m data set (Oliphant, 2017). The managed forest class was mapped by capturing areas of forest change from 2001–2016 via the Global Forest Change (GFC) 30‐m product (Hansen et al., 2013). The natural forest class was mapped using the Landsat Vegetation Continuous Fields product (Sexton et al., 2013). Topographic depressions were determined based on surface curvature and flow accumulation calculated using the Shuttle Radar Topography Mission (SRTM) 1‐arc second digital elevation models (DEM). These six classes were combined into a single map hierarchically based on the order priority mentioned above. Specifically, the classification began with the class with the highest priority (water) and continued following the order of priority. If a pixel had already been assigned a class value with higher priority, it would no longer be eligible for subsequent classification. All remaining pixels that were not mapped into any of the first six classes were classified into either shrub/grass or bare ground, based on the Landsat‐derived Normalized Difference Vegetation Index (NDVI) with a threshold value of 0.5 (shrub/grass: >0.5, bare ground: ≤0.5). Though all of the above classes were mapped, analysis was conducted only on those classes which correspond with the self‐reported LU survey (croplands, managed forests, and natural forests) or are known to be relevant to mosquito breeding (topographic depressions) (Rosenberg, 1982).

The resultant map was assessed for accuracy using the assessment methodology based on the fuzzy set theory developed by Woodcock and Gopal (2000). The total weighted accuracy for the map is 81.73% when fuzziness (i.e., tolerance for error) is considered. For the LCs of primary interest, natural forests, managed forests, and croplands, the accuracies are 90%, 48%, and 84%, respectively. We were unable to assess the accuracy of the topographic depression class due to a lack of ground truth data (see Appendix). The full results of the accuracy assessment can be found in Table A3.

For each village surveyed, we derived a satellite‐based characterization of village environmental settings by calculating the area of each mapped LCLU category within a 2‐km radius of the center of the village. The rationale of choosing 2 km as our buffer distance is grounded in the flight dispersal of the major malaria vectors in the region and the size of the villages. *An*. *minimus* and *An*. *dirus* have estimated flight ranges of 1 and 2 km, respectively (Dev et al., 2004; Marchand et al., 2004). Villages A, B, and C are quite small, with homes and infrastructure covering less than 0.5 km^2^. However, Villages D and E are larger and less isolated than the other villages, while also being relatively close to each other. A 2‐km buffer proved the most reasonable to allow for maximal coverage of the LC types that villagers likely spend the majority of their time in for all villages, while also limiting the overlap between Villages D and E (approximately 5 km^2^ overlap exists between buffers).

We also analyzed forest cover change. We calculated the area of forest loss within 2 km of each village for the year 2016 (the year of survey data collection) derived from the GFC 30‐m product described above. We also calculated the annual rate of forest loss for the previous 5 years (2012–2016) and the total area of forest loss within that period.

### 2.3 Statistical Analysis

Of the 990 participants, 11 were excluded due to missing age (*n* = 5) or unknown pregnancy status (*n* = 6). Univariate and multivariate logistic regression analysis was chosen to understand the association between the exposure variables and outcome (presence of malaria parasites in the blood sample). Due to the low overall prevalence of malaria (9.40%, *n* = 92), *P*. *falciparum*, *P*. *vivax*, and mixed infections were all considered as a positive case. Univariate analysis was performed to assess the relationship between individual malaria and each subset of exposure variables, including self‐reported LU (frequent visits to farms, forests, and plantations), satellite‐based environmental village settings (area of croplands, natural forests, managed forests, and depressions in square kilometers), and forest cover change variables (area of recent forest loss, rate of forest loss, and area of 5‐year cumulative forest loss). The variables of frequent visits to refugee camps or mines were not analyzed because no participants responded affirmatively to those questions.

For each univariate model where the assessed exposure variable was found to be significantly associated with malaria (*p* < 0.05), potential confounders were progressively added (age, age squared, sex, pregnancy status, travel status, and family travel status). Biologically plausible confounders found to be significant remained in the final adjusted model while nonsignificant variables were removed. Within the fully adjusted models, interactions by sex were examined via interaction terms. If interactions were significant, stratified models were considered. Odds ratios (ORs) and 95% confidence intervals (CIs) were calculated for the resultant adjusted and stratified models. All data analysis was undertaken in R statistical software packages.

Finally, we conducted three sensitivity analyses to evaluate the robustness of our findings. Sensitivity Analysis I sought to examine Village D's impact on the results by removing Village D from the study sample and comparing these results to those of the main analysis. The respondents from Village D were all working‐aged males—very different than the respondent demographics from the other villages, which more closely follow the general demographics of Myanmar (MPHC, 2014). Sensitivity Analysis II assessed the influence of village environmental settings on malaria infection in those participants who reported no to frequently visiting farms, forests, or plantations (i.e., the participants answered “no” to every LU question; see section 2.3) and presumably primarily remained in the village during the past six months. Sensitivity Analysis III was conducted to evaluate further the relationship found between forest loss metrics and malaria. In this analysis, we removed Village C from the data set because the amount of forest loss surrounding that village was significantly higher than for the other four villages (section 3.5 and Figure 5).

## 3 Results

### 3.1 Demographics: Confounders and Effect Modifiers

As shown in Table 1, 9.4% (*n* = 92) of our study population tested positive for malaria parasites via usPCR testing. *P*. *vivax* malaria is more prevalent in this region: 30.4% (*n* = 28) tested positive for *P*. *falciparum*,while 63.0% (*n* = 58) tested positive for *P*. *vivax*, with the remaining 6.5% (*n* = 6) identified as mixed infections. Nearly every positive case was asymptomatic/subclinical. While debate exists regarding the confirmed definition of asymptomatic malaria, the most widely used criteria are the presence of parasites in peripheral thick blood smears, an axillary temperature <99.5 °F, and no evidence of malaria‐related symptoms (Laishram et al., 2012). Of the 92 positive cases, only one case reacted positively to RDT testing. No respondent had a fever at the time of data collection, though a few respondents claimed to have had a fever within the past 24 hr (*n* = 4) or past 2 weeks (*n* = 6). Similarly, low numbers were reported for other symptoms experienced in the past 2 weeks, including headache (*n* = 34), body aches (*n* = 31), nausea (n = 3), vomiting (*n* = 5), abdominal pain (*n* = 15), loss of appetite (*n* = 5), and fatigue (*n* = 6).

Our sample population skewed male (60.4%) because participants from Village D were all male. Otherwise, each village was split approximately equally between male and female participants. Sex was not determined to be a significant confounder within our study; however, a significant interaction was evident between sex and farmland use. Therefore, models stratified by sex are presented for farm‐related variables below.

**Figure 2:**
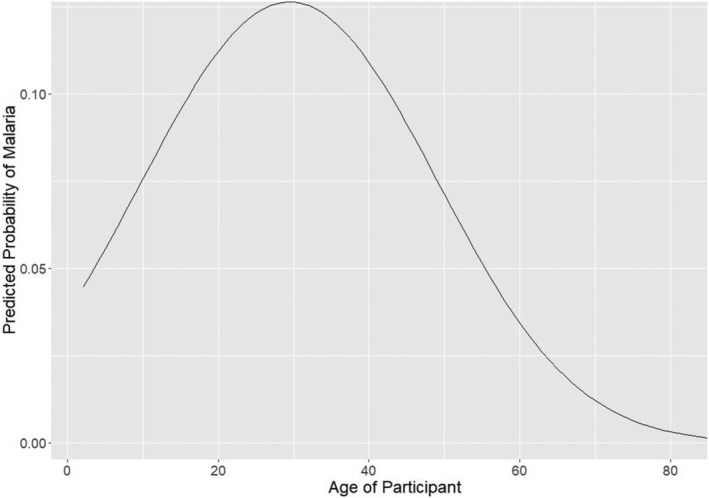
Logistic regression univariate model derived relationship between malaria risk and age.

The participant age distribution generally follows the age distribution of Myanmar (MPHC, 2014), with slightly more participants in the 25–54 age group (52.2%) when compared to the country overall (42.51%) and slightly less in all other groups. This is again due primarily to Village D, where the sample is composed entirely of working‐aged males. A nonlinear relationship between age and probability of malaria infection was discovered (Figure 2). Age and age squared proved to be significant confounders and are therefore included in each adjusted model presented.

Six women reported being pregnant during the time of sample collection. However, despite low overall malaria prevalence, malaria infection within the pregnant cohort was high at 50% (*n* = 3). For this reason, pregnancy status is included in all fully adjusted models presented and within any female stratified models.

For travel status, 463 (47.3%) participants reported traveling outside the village within the past 6 months, with the majority of those (*n* = 198) reporting from Village D. Mobility of family members (although not necessarily the participants themselves) was nearly equally high: 390 (39.8%) participants reported having a family member from their household traveling outside the village within the past 6 months. Neither of these variables was determined to be significantly associated with malaria infection through univariate logistic regression and were therefore not adjusted for in the final models.

### 3.2 Sensitivity Analysis Demographics Comparison

The sensitivity analyses altered the demographics of our study sample in different ways (Table 2). The exclusion of Village D (Sensitivity Analysis I) resulted in demographics more similar to the overall demographics of Myanmar (MPHC, 2014), in terms of both age and sex distribution. Roughly half of the Primary Analysis group answered “No” to frequenting farms, forests, or plantations. When these respondents were grouped for Sensitivity Analysis II, the age distribution skewed much younger, with very few children removed from the sample. Sensitivity Analysis III was conducted to investigate the link to forest loss; therefore, the exclusion of Village C had less to do with demographics and more to do with the landscape of the village, which is further explained in section 3.5; however, the changes in demographics are shown here.

**Table 2:** Demographic Information of Primary Analysis and Sensitivity Analyses I, II, and III

Village	Primary analysis	Sensitivity analysis I (village D excluded)	Sensitivity analysis II (answered no to all three LU questions)	Sensitivity analysis III (village C excluded)
Population sampled (*n*)	979	780	492	787
Sex: *n* (% of sample population used in analysis)				
Female	388 (39.6%)	388 (49.7%)	233 (47.3%)	295 (37.5%)
Male	591 (60.4%)	392 (50.3%)	259 (52.6%)	492 (62.5%)
Age: *n* (% of sample population used in analysis)				
0–14	233 (23.8%)	233 (29.9%)	215 (43.7%)	153 (19.4%)
15–24	152 (15.5%)	128 (16.5%)	81 (16.5%)	118 (15.0%)
25–54	511 (52.2%)	341 (43.7%)	166 (33.7%)	446 (56.7%)
55–64	49 (5.0%)	44 (5.6%)	10 (2.0%)	43 (5.5%)
65+	34 (3.5%)	34 (4.4%)	20 (4.1%)	27 (3.4%)
Pregnancy status: *n* (% of sample female population used in analysis)				
Pregnant	6 (1.5%)	6 (1.5%)	4 (1.7%)	6 (2.0%)
Not pregnant (excludes males)	382 (98.5%)	382 (98.5%)	229 (98.2%)	289 (98.0%)
Travel: *n* (% of sample population used in analysis)				
Participant traveled outside village in past 6 months	463 (47.3%)	265 (34.0%)	186 (37.8%)	411 (52.2%)
Family member(s) of participant traveled outside village in past 6 months	390 (39.8%)	351 (45.0%)	216 (43.9%)	307 (39.0%)
Self‐reported exposure: *n* (% of sample population used in analysis)				
Frequently visits forest areas	249 (25.4%)	174 (22.3%)	0 (0%)	225 (28.6%)
Frequently visits plantations	203 (20.7%)	94 (12.1%)	0 (0%)	179 (22.7%)
Frequent visits farms	283 (28.9%)	269 (34.5%)	0 (0%)	192 (24.4%)
Did not select any land use option	492 (50.3%)	418 (53.6%)	492 (100%)	393 (50.0%)
Malaria prevalence: *n* (% of sample population used in analysis)				
*P*. *falciparum* mono	28 (2.9%)	27 (3.5%)	7 (1.4%)	15 (1.9%)
*P*. *vivax* mono	58 (5.9%)	46 (6.3%)	29 (5.9%)	36 (4.6%)
Mixed *P*. *falciparum* and *P*. *vivax*	6 (0.6%)	6 (0.8%)	2 (0.4%)	2 (0.3%)
Any malaria	92 (9.4%)	79 (10.1%)	38 (7.7%)	53 (6.7%)

### 3.3 Self‐Reported Use of Landscape

Fully adjusted models (adjusted for age, age squared, and pregnancy) were created for each of the self‐reported use of landscape variables, and ORs were calculated (Table 3). Frequent visits to a forest were not associated with malaria (OR: 1.26, 95% CI: 0.76–2.04). However, frequent visits to a plantation were found to be protective for malaria (OR: 0.51, 95% CI: 0.27–0.92). The association between frequent visits to a farm and malaria was modified by sex; therefore, we present fully adjusted models stratified by sex. Within stratified models, we observed no relationship between frequent visits to a farm and malaria for females (OR: 1.36, 95% CI: 0.57–3.26), but a strong positive relationship was observed among males (OR: 3.86, 95% CI: 2.13–7.06). These results were reinforced by Sensitivity Analysis I, except for frequent plantation visits no longer being significantly protective.

**Table 3:** Model Results Expressing the Risk of *Plasmodium* Presence in the Blood as a Function of Self‐Reported LU Visit Frequency

	Primary analysis		Sensitivity analysis I (village D excluded [all male village])	
Variable	OR (95% CI)	*p* value	OR (95% CI)	*p* value
Frequently visits forest areas	1.26 (0.76–2.04)	0.3571	1.29 (0.73–2.21)	0.3683
Frequently visits plantations	0.51 (0.27–0.92)	0.0318	0.58 (0.24–1.22)	0.1820
Frequently visits farms—female	1.36 (0.57–3.26)	0.4794	1.11 (0.36–3.59)	0.8605
Frequently visits farms—male	3.86 (2.13–7.06)	< 0.001	2.74 (1.46–5.25)	0.0019

*Note*. Blue cells indicate protective associations, red cells indicate risk associations, while white cells indicate nonsignificant associations. When printed in gray scale, significant associations (both risk and protective) appear shaded, while nonsignificant associations do not.

Respondents were able to select more than one self‐reported LU option. While 253 individuals selected only a single LU and 492 selected no LU options, 215 selected two options (i.e., forests and plantations or farms and forests), and 15 selected all three options. Univariate analyses of variables created to capture the relationships between multi‐LU respondents were not found to be significant and therefore were not further investigated. Interestingly though, 4 of the 15 (26%) participants who responded yes to all three LU options tested positive for malaria.

### 3.4 Village Environmental Settings

The results of the satellite‐based LCLU mapping revealed large swaths of natural forests and croplands dominating the surrounding landscapes of the study villages within the 2‐km buffer. All village landscapes contained some area of managed forests (between 2% and 7% of the total area mapped), but Village C was distinctive in that 24% of its landscape was covered by managed forest (Table 4). In comparison, depressions covered a very small percentage of the surrounding landscape (between 0.2% and 0.6% of total area mapped) (Table 4).

**Table 4:** Areas of Relevant LCLU Classes (Percentage of Total Land) Within 2 km of a Village Center in Square Kilometer

Village	A	B	C	D	E
Croplands	1.58 (12.6%)	5.90 (47.0%)	0.26 (2.1%)	1.89 (15.1%)	3.09 (24.5%)
Managed forests	0.74 (5.9%)	0.84 (6.7%)	2.97 (23.6%)	0.67 (5.4%)	0.28 (2.3%)
Natural forests	7.19 (57.2%)	2.96 (23.5%)	8.14 (64.8%)	4.03 (32.1%)	1.53 (12.2%)
Depressions	0.08 (0.6%)	0.03 (0.2%)	0.04 (0.3%)	0.06 (0.5%)	0.06 (0.5%)

**Figure 3:**
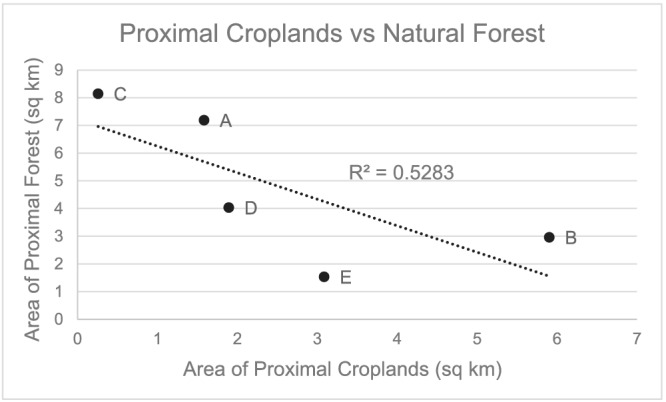
Relationship between the area of natural forest and croplands among the villages.

**Figure 4:**
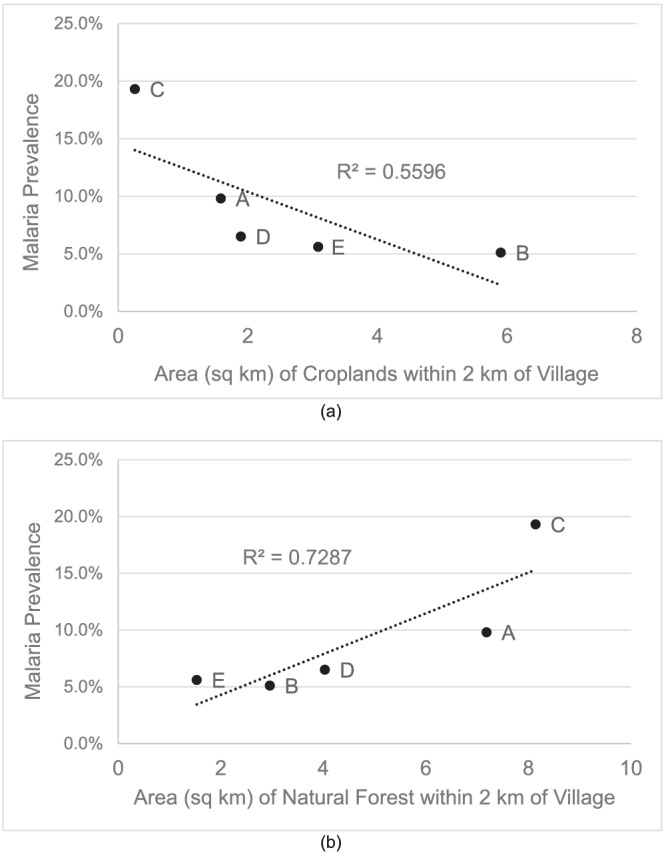
Relationships between malaria village prevalence and (a) area of croplands within 2 km of a village and (b) area of natural forest within 2 km of a village.

An inverse relationship between the area of natural forest and croplands was observed among the villages (Figure 3). Villages with expansive areas of natural forest (Villages A, C, and D) had comparatively small areas of croplands. Villages with large areas of croplands similarly have less area of natural forest (Villages B and E). A weak negative correlation was found between proximal croplands and village‐level malaria prevalence (*R* ^2^ = 0.56) (Figure 4a), while a positive correlation was found between proximal natural forest and village‐level malaria prevalence (*R* ^2^ = 0.73) (Figure 4b).

Fully adjusted models assessed the relationship between malaria and each of the village environmental settings variables of interest. Area of natural forest within 2 km of a participant's village and area of managed forest within 2 km of a participant's village were found to be strongly associated with increased risk of malaria (OR: 1.96, 95% CI: 1.60–2.41, and OR: 1.35, 95% CI: 1.23–1.50, respectively). We found that sex modified the relationship between malaria and area of croplands within 2 km of a participant's village, and thus, we present stratified results. Among females, proximal croplands were found to be strongly protective (OR: 0.52, 95% CI: 0.37–0.69), whereas a much weaker suggestive protective effect was observed among males (OR: 0.82, 95% CI: 0.67–1.00) (Table 5). The results were reinforced by Sensitivity Analysis II, which only analyzed data on study participants that did not claim to frequently visit any of the three LCs investigated (demographics for Sensitivity Analysis II compared to the Primary Analysis can be found in section 3.2 and Table 2). No significant association was found between malaria and areas of topographic depressions.

**Table 5:** Model Results Expressing the Risk of *Plasmodium* Presence in the Blood as a Function of Village Proximal LC

	Primary analysis		Sensitivity analysis II (answered no to all three LU questions)	
Variable	OR (95% CI)	*p* value	OR (95% CI)	*p* value
Area of natural forest	1.96 (1.60–2.41)	<0.001	3.09 (2.12–4.63)	<0.001
Area of managed forest	1.35 (1.23–1.50)	<0.001	1.50 (1.27–1.81)	<0.001
Area of croplands—female	0.52 (0.37–0.69)	<0.001	0.47 (0.29–0.69)	0.0007
Area of croplands—male	0.82 (0.67–1.00)	0.0576	0.32 (0.14–0.64)	0.0046
Area of depressions	0.004 (0.002–1,411.04)	0.3950	0.00002 (0.00–15,821.00)	0.3178

*Note*. Blue cells indicate protective associations, red cells indicate risk associations, while white cells indicate nonsignificant associations. When printed in gray scale, significant associations (both risk and protective) appear shaded, while nonsignificant associations do not.

### 3.5 Forest Loss

**Figure 5:**
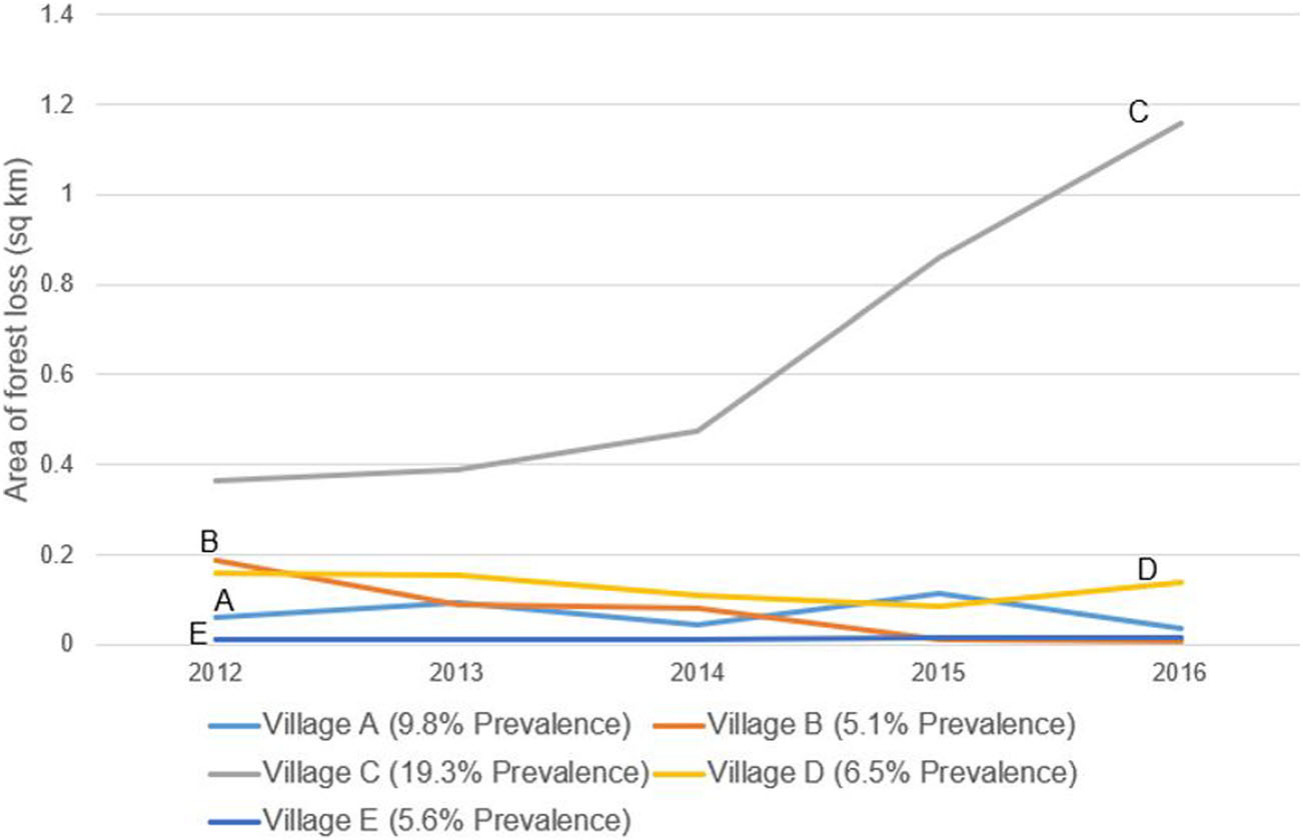
Annual area of forest loss in square kilometer within 2 km of each village over the 5 years preceding the survey data collection. Village‐level malaria prevalence included in the key.

**Figure 6:**
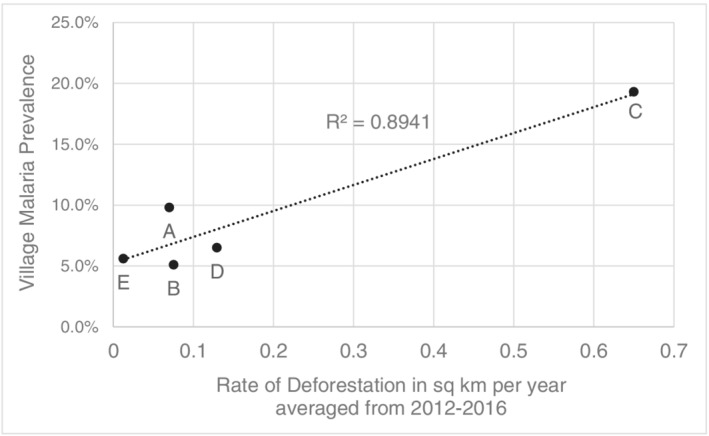
Relationship between village‐level malaria prevalence and the average rate of deforestation (km^2^/year) over the 5 years preceding survey data collection.

Each village exhibited very different patterns of forest cover change. While all have experienced some amount of forest loss, Village C lost by far the highest amounts of forest in the year of data collection (2016) and the 5 years preceding our survey (Figure 5). When compared to village‐level malaria prevalence, a high positive correlation was found between malaria prevalence and the rate of deforestation surrounding a village (km^2^/year) (Figure 6). This was further corroborated by the fully adjusted models, which found that recent (2016) deforestation (OR: 3.97, 95% CI: 2.57–6.13), rate of deforestation (OR: 14.30, 95% CI: 6.20–32.99), and total deforestation over 5 years (OR: 1.70, 95% CI:1.44–2.01) were all strongly associated with increased malaria risk. However, these results were not reinforced by Sensitivity Analysis III, wherein none of the relationships remained significant when Village C was removed (Table 6).

**Table 6:** Model Results Expressing the Risk of *Plasmodium* Presence in the Blood as a Function of Village Proximal Deforestation

	Primary analysis		Sensitivity analysis III (village C removed)	
Variable	OR (95% CI)	*p* value	OR (95% CI)	*p* value
Area of deforestation (2016) (km^2^)	3.97 (2.57–6.13)	<0.001	0.19 (0.00–37.64)	0.5503
Rate of deforestation (2012–2016) (km^2^/year)	14.30 (6.20–32.99)	<0.001	1.77 (0.003–1228.7)	0.8616
Total deforestation (2012–2016) (km^2^)	1.70 (1.44–2.01)	<0.001	1.12 (0.31–4.15)	0.8616

*Note*. Blue cells indicate protective associations, red cells indicate risk associations, while white cells indicate nonsignificant associations. When printed in gray scale, significant associations (both risk and protective) appear shaded, while nonsignificant associations do not.

## 4 Discussion

The challenge posed by asymptomatic reservoirs for malaria elimination is illustrated by this study's findings that only 1 out of the 92 malaria cases identified was detected by the standard test used for routine surveillance and clinical diagnosis. Routine testing found a prevalence of just 0.1% across five villages, while more sensitive molecular testing uncovered villages with up to more than 20% prevalence of low‐density malaria infections. No cases exhibited signs of fever at the time of data collection, and very few reported any recent physical symptoms. Although data are limited, a growing body of evidence highlights the critical importance of all malaria infections, symptomatic or asymptomatic, in continued and sustained transmission. This study further highlights the growing need to identify these infection reservoirs for targeted interventions with a method not solely reliant on routine testing and self‐reporting.

One such criterion that should be considered when implementing a targeted elimination strategy is the amount of natural forest cover surrounding a village. Our findings further corroborate the association between malaria risk and forest cover found in other studies (Tipmontree et al., 2009; Zaw et al., 2017). While previous work has established a link between forest workers and malaria risk, our research expands upon this by revealing that persons living in villages where the dominant LC is natural forest are at an increased malaria risk, regardless of whether or not they work or spend time in forested areas. This is further supported by our subsample of participants who did not claim to frequently visit a forested area (Sensitivity Analysis II) but experienced a high positive association with malaria infection relating to the area of natural forest coverage near their village.

Conversely, persons living in villages with high areas of croplands experience decreased malaria risk, unless they are explicitly working/frequenting those areas. This is especially true for men, for whom our results indicate a substantial increase in risk for frequent farm visitors but a strong protective effect for men who merely live close to large areas of croplands (Sensitivity Analysis II) but do not frequently visit those lands. We do not believe that proximal croplands are necessarily “protective” for malaria. Instead, we surmise that the protective effect of croplands is in reality due to the minimizing of the “riskier” LC, natural forest. We found that villages with high areas of proximal croplands also have comparatively low areas of proximal natural forest (section 3.4 and Figure 3). Villages with higher percentages of croplands than natural forests, Village B (3% forest, 47% cropland) and Village E (12% forest, 25% cropland), have the lowest rates of malaria at 5.1% and 5.6%, respectively. Similarly, areas with higher percentages of natural forest than croplands, Village A (57% forest, 13% cropland) and Village C (65% forest, 2% cropland), have the highest village‐level prevalence of malaria at 9.8% and 19.3%, respectively. One potential explanation for this relationship may have to do with the dominant vector species in Ann Township. *An*. *dirus*—a forest‐dwelling species—has a longer flight range (~2 km) than the other dominant malaria vector in Ann Township—*An*. *minimus*, which prefers lowland areas and has a flight range half as long (~1 km) (Dev et al., 2004; Marchand et al., 2004). Therefore, the forest‐dwelling *An*. *dirus* is more easily able to reach persons living close to its natural habitat, whereas *An*. *minimus* is less likely to travel far enough to bite anyone that does not explicitly visit its habitat (i.e., cropland).

The observed differences between the sexes in farm work‐associated risk were an unexpected finding but could be explained by the work‐related gender dynamics of the villagers. While women who report frequenting farms do not experience an increase in risk, there is a substantial increase in risk for men (OR: 3.86, 95% CI: 2.13–7.06). Akter et al. (2017) found that men and women share many of the same roles in rice farming. However, some roles are gendered, with men participating more in land preparation and fertilizer/pesticide application and women participating more in seedling transplanting and food preparation for laborers. This gendered dynamic to land preparation could result in males spending comparatively longer periods in the fields, though we currently have no evidence of this. Although it is also possible, and semisupported by our data, that men in this region often take on multiple livelihood roles. Within our sample, 32% of women and 27% of men reported frequenting a farm, indicating that slightly more women participate in farm work than males. However, 53% of men who reported frequenting a farm also reported frequenting a forest, compared to only 31% of the women who frequented farms. Therefore, men who farm are also more likely to frequently engage in other activities that could increase their risk of malaria exposure. While engagement in multiple LUs was not found to be a significant risk factor within our data set, it does appear to be worthy of further study with a more sensitive survey methodology.

The interactions between frequenting plantations and managed forest LC are essentially the inverse of the farm/cropland discussion above. Frequent visits to a plantation were found to be a protective factor, while in contrast, high areas of plantation LC surrounding a village increased risk. Literature indicates that plantation jobs (fruit, rubber, and teak) in Southeast Asia typically increase risk (Singhasivanon et al., 1999). This is in‐line with our results on the amount of proximal managed forest coverage. Indeed, Village C displays the highest proportion of managed forest (24%) and also the highest prevalence of malaria (19.3%). Interestingly, the villages with the lowest proportions of managed forest, Village D (5.4%) and Village E (2.3%), counterintuitively report the highest numbers of respondents saying they frequent plantations (*n* = 109 and *n* = 67, respectively). Village C, in comparison, had only 24 respondents frequenting plantations. Overall, malaria prevalence rates in Villages D and E were low (6.5% and 5.6%, respectively). When viewed via satellite imagery, Villages D and E appear much less isolated than the other villages, both being relatively close to the only airport within Ann Township and having much higher percentages of human infrastructure than the rest of the villages. Second, Villages D and E are very close to each other. Approximately 5 km^2^ of overlapping area exists between the 2‐km buffers created for the villages. This leads us to believe that there is a confounding variable influencing the plantation workers from these two villages that we were unable to capture in our study.

While the static LCLU mapped and calculated for this study revealed interesting relationships, LCLU is rapidly changing across Myanmar as the economy grows and expands. One of the most prominent areas of change is forest loss, with estimates of nearly 2 × 10^6^ ha of intact forest lost occurring annually (Bhagwat et al., 2017). Since we discovered that both proximal natural forest and managed forest LC increased malaria risk, it seemed logical that any significant changes to those LC types could also influence malaria risk. We identified strong associations between multiple different metrics of forest cover loss and malaria risk. However, Village C has experienced a significantly higher amount of forest cover loss than any of the other villages, which profoundly influenced the results. No significant associations were identified when Village C was removed from the analysis. We believe, though, that Village C is less of an outlier and instead represents a different type of village than the others included in our study. Preliminary results from ongoing projects in the region (data not presented here) indicate that other villages are experiencing similar levels of forest cover loss and high prevalence of malaria, though more research is necessary for this area.

Some limitations of this research include the moderate resolution of the satellite imagery and potential confusion in questionnaire responses. Finer spatial resolution satellite data would have been welcome, particularly in identifying managed forest cover, for which our mapped accuracy was only 48%, and topographic depressions. While SRTM data have been successfully used in other studies to identify topographic depressions which may serve as mosquito breeding habitats (Clennon et al., 2010), our inability to assess the accuracy of this class without extensive ground truth data led to it being ranked low within the hierarchical mapping scheme, which likely resulted in an underestimation of these areas. However, the goal of this research was to identify criteria that could be used easily to locate reservoirs of malaria. In essence, fine spatial resolution LCLU mapping is more time consuming, complicated, and costly than moderate resolution mapping. The mapping methodology presented here relies on freely available public data and can be easily replicated for other locations and dates.

Within our questionnaire, it is unclear if our definitions of LU match the respondents' perceptions. For example, we intended for frequent visits to a farm to mean a rice paddy or large cropped field. However, it is possible that for our respondents, subsistence agricultural plots near to their homes could be considered as farms. It is also possible that farming in our respondents' interpretation refers to forest‐related work (Zaw et al., 2017) or foraging for wild vegetables in forested areas (Cornish & Ramsay, 2018). These potential differences in definition could impact the interpretation of our results. However, the results gleaned from the remotely sensed LC variables are less vulnerable to misinterpretation, which helped contextualize the questionnaire results.

## 5 Conclusions

As malaria transmission declines, targeted interventions will become the highest priority to malaria elimination in Southeast Asia. Our study found that the overwhelming majority of malaria cases in Ann Township are asymptomatic, rendering them invisible to in situ rapid diagnostic techniques and missed by the reporting mechanisms currently in place for symptomatic treatment‐seeking individuals. Considering the highly heterogeneous and rapidly changing prevalence of asymptomatic malaria identifying areas to target is growing in difficulty. By pairing remotely sensed indices with survey data, we were able to contextualize LCLU metrics to form a cohesive picture of malaria within Ann Township that can bolster elimination efforts. Primarily, villages with high natural or managed forest cover in their immediate proximity are the locations where one is most likely to find persons with malaria, with considerations for age and pregnancy status. For villages with large areas of croplands, prevention strategies should consider focusing on men, particularly those working on farms.

More research is needed to assess the causal link between forest cover and malaria in Myanmar. While we hypothesize that it may have something to do with the LC preferences of the two dominant mosquito species, entomological data for the region are sparse. Without more information on this link, it will be difficult to ascertain how forest cover change will influence malaria infections for the region. As the economy of Myanmar grows, likely, the conversion of natural forests to teak, rubber, or other plantations will accelerate. Understanding the relationship between malaria and deforestation and forest conversion will be critical to eliminating malaria under these rapidly changing socioeconomic conditions.

Now more than ever, models that allow for the identification of likely reservoirs of malaria, but are disconnected from symptomatic carriers seeking treatment or broad coverage in situ rapid diagnosing, are needed to proceed forward with targeted interventions. Remote sensing offers a means to quickly locate areas that meet the LC criteria discussed and provides promising data and methodologies to explore other LCLU that may influence malaria risk.

## Conflict of Interest

The authors declare no conflicts of interest relevant to this study.

## Data Availability

Field survey data are protected per Duke University Institutional Review Board policy. The 30‐m LCLU map is available through the PANGAEA data repository https://doi.org/10.1594/PANGAEA.921126 (Chen et al., 2020). Forest Cover Change data are available through http://earthenginepartners.appspot.com/science‐2013‐global‐forest (Hansen et al., 2013).

## Appendix A Accuracy Assessment of 30 m LCLU Classification

### A.1 Determining the Sample Size and Allocation per Strata

For this study, the final number of reference samples was 833. The distribution of samples among strata (i.e., LCLU classes) was between proportional allocation and equal allocation. We specified a minimum sample size per class to be 75 samples to ensure adequate sampling for rare classes (e.g., villages). The spatial unit of analysis was a 30‐m by 30‐m pixel, consistent with the spatial unit of the LCLU classification. The final allocation of samples per strata is summarized in Table A1 below.

**Table A1:** Reference Samples, by Strata

Strata	Area (pixels)	Area (proportion)	Number of samples
Perennial water	300,875	4.30%	75
Impervious surface	6,876	0.10%	75
Villages	5,315	0.10%	75
Croplands	246,172	3.50%	75
Managed forests	394,159	5.70%	75
Natural forests	5,669,060	81.70%	308
Shrub and grass	262,724	3.80%	75
Bare surfaces	53,672	0.80%	75
Total	6,938,853	100.00%	833

### A.2 Expert Sample Review

Gopal and Woodcock (1994) developed foundational methods for accuracy assessment of map classification (that uses crisp sets) and reference classification that uses fuzzy sets. Woodcock and Gopal (2000) applied this method to a forest cover classification study. In their method, they propose a five‐level linguistic scale to evaluate the correctness of map classification accuracy for each sample. The highest level to the lowest level of correctness is “absolutely right,” “good answer,” “reasonable or acceptable answer,” “understandable but wrong,” and “absolutely wrong,” respectively (Woodcock & Gopal, 2000).

Table A2 reproduces the linguistic‐measurement scale developed by Woodcock and Gopal (2000). We applied this scale in this study to assess the accuracy of the LCLU mapping. Four expert interpreters evaluated the 833 samples against the linguistic‐measurement scale. The samples were compared with Google Earth imagery and responses were recorded using Microsoft Access.

**Table A2:** The Linguistic‐Measurement Scale Adapted From Woodcock and Gopal (2000)

Rank	Description
5	Absolutely right: No double about the match. Perfect.
4	Good answer: Would be happy to find this answer given on the map.
3	Reasonable or acceptable answer: Maybe not the best possible answer but it is acceptable; this answer does not pose a problem to the user if it is seen on the map.
2	Understandable but wrong: Not a good answer. There is something about the site that makes the answer understandable but there is clearly a better answer. This answer is a problem.
1	Absolutely wrong: The answer is absolutely unacceptable and completely wrong.

### A.3 Calculation of Accuracies

Two operators developed by Woodcock and Gopal (2000), “MAX” and “RIGHT,” are summarized in Table A3. These metrics provide information on the distribution of errors and also the frequency of errors:

1. MAX: “Highest rating given to a category for a given site to measure a match and provides a conservation estimate of accuracy”
2. RIGHT (R): “Accepts matches using any degree of right, which in the linguistic scale used here is any score greater than or equal to 3”

**Table A3:** Results of MAX and RIGHT Functions

Map classes	Number of samples	Max (rank = 5)	RIGHT (rank ≥3)	Area weights (proportion)
Perennial water	75	21	26	0.043
Impervious surface	75	6	36	0.001
Villages	75	44	61	0.001
Croplands	75	51	63	0.035
Managed forests	75	22	36	0.057
Natural forests	308	229	277	0.817
Shrub and grass	75	1	15	0.038
Bare surfaces	75	0	17	0.008
Total	833	374	531	
